# High awareness, low compliance: rabies knowledge and dog ownership practices among pastoralists, Marsabit County, Kenya

**DOI:** 10.3389/fvets.2025.1682727

**Published:** 2025-11-26

**Authors:** Pauline N. Gitonga, Brian Ogoti, Teresa Lopuwa Etapar, Andre Coetzer

**Affiliations:** 1 Department of Biomedical Sciences, Colorado State University, Fort Collins, CO, United States; 2 Centre for Epidemiological Modelling and Analysis (CEMA), The University of Nairobi, Nairobi, Kenya; 3 ASAL eXtension, Nairobi, Kenya; 4 Department of Medical Microbiology and Immunology, The University of Nairobi, Nairobi, Kenya; 5 Veterinary Paraprofessional Intern, Department of Agriculture, Livestock and Fisheries Development, Marsabit, Kenya; 6 Global Alliance for Rabies Control, Manhattan, KS, United States

**Keywords:** rabies, knowledge-practice gap, pastoralists, dog ownership, Kenya

## Abstract

**Introduction:**

Rabies is a fatal yet preventable zoonotic disease that disproportionately affects underserved communities in endemic regions. Understanding community-level Knowledge, Attitudes, and Practices (KAP) is essential for designing effective rabies control programs, particularly in remote pastoralist settings where access to healthcare and information is limited. This study assessed community knowledge, attitudes, and practices regarding rabies and dog ownership among pastoralist communities in Marsabit County, Kenya. It aimed at evaluating levels of rabies knowledge and dog care practices across demographic groups to identify gaps that could inform locally appropriate prevention strategies.

**Methods:**

A cross-sectional KAP survey was conducted in May 2023 among 411 households using a structured questionnaire, with stratified random sampling employed to ensure village-level representation.

**Results:**

Overall, 86.1% of respondents demonstrated adequate knowledge of rabies, with slightly higher knowledge among dog owners (87.0%). However, only 18.8% of dog owners met the threshold for responsible ownership. Among dog owners, rabies vaccination coverage was just 22%, highlighting a significant knowledge-practice gap. Willingness to pay for dog sterilisation surgery was a significant predictor of rabies knowledge (aOR 2.95, 95% CI: 1.33–7.22, *p* = 0.0110), while gender was the only significant predictor of responsible dog ownership, with females having lower odds (aOR 0.50, 95% CI: 0.25–1.02, *p* = 0.0495).

**Discussion:**

Despite high levels of rabies knowledge, preventive practices such as vaccination, deworming, and responsible dog ownership remain suboptimal in Loiyangalani town. A multifaceted, community-based approach is urgently needed to close the knowledge–practice gap and advance rabies elimination goals in remote pastoralist communities.

## Background

Rabies is a vaccine-preventable viral zoonosis responsible for an estimated 59,000 human deaths each year, especially in resource-limited rural communities in Africa and Asia where the disease burden is highest (1, 2). Rabies is transmitted through exposure to the infectious saliva of infected animals, most commonly via bites or scratches, with domestic dogs accounting for over 99 percent of human rabies cases globally (3). Despite being almost always fatal once clinical symptoms appear, it is entirely preventable through the timely administration of Post Exposure Prophylaxis (PEP), which includes immediate and thorough wound cleaning, the administration of a series of rabies vaccinations, and, when indicated, local wound infiltration with rabies immunoglobulin (RIG) (4).

The World Health Organisation (WHO) recommends vaccinating at least 70% of at-risk dogs to secure herd immunity. This target is the cornerstone of the global “Zero by 30” strategy, a concerted effort by WHO, World Organisation for Animal Health (WOAH), and Food and Agriculture Organisation (FAO) to eliminate human deaths from dog-mediated rabies by 2030. Kenya’s stepwise strategic plan for rabies control is anchored in this tripartite global goal (5).

Rabies control in endemic areas mainly depends on stopping disease transmission among dog populations through large-scale vaccination campaigns (6). However, achieving this remains difficult in many areas, especially where dogs are free-roaming, owned but roaming, or community-owned, which makes them hard to access during vaccination efforts. Besides variations in dog ownership, behavioural and cultural factors greatly affect the success of rabies control measures (6). This is particularly true in pastoralist communities with high mobility, limited access to veterinary services, and dogs playing both utilitarian and cultural roles. To bridge the gap in formal veterinary care in these underserved regions, community-based personnel, locally known as Community Disease Reporters (CDRs), are utilised in Kenya. CDRs are frontline community workers tasked with disease search, reporting, and basic health extension, aligning with the principles for Community Animal Health Workers (CAHWs) endorsed by organisations like WOAH (7). The risk of rabies transmission increases due to close human-animal interactions, low awareness of rabies prevention, and limited knowledge of responsible dog care practices (8).

In Marsabit County, Kenya, pastoralist communities in and around Loiyangalani Town have been grappling with recurring suspected and confirmed rabies cases. The County Directorate of Veterinary Services (9) has documented this concerning trend. Previous dog population control efforts, such as mass culling using strychnine, were rightfully discontinued due to ethical, ecological, and public health concerns. In response, the county government intended to implement a humane and sustainable rabies control strategy, including a mass vaccination and high-volume sterilisation campaign scheduled for November 2023.

Past studies have shown that interventions that do not consider local beliefs, practices, and socioeconomic constraints tend to yield suboptimal outcomes (10, 11), it was essential to first understand the baseline Knowledge, Attitudes, and Practices (KAP) related to rabies and dog ownership within the pastoralist communities, a population historically overlooked in rabies research in Kenya and beyond. While KAP studies on rabies are common, few have focused specifically on remote, nomadic populations such as those living in and around Loiyangalani town.

This unique sociocultural and ecological context introduces distinct challenges and opportunities for disease prevention, making it a critical, yet underexplored setting, for such research. Accordingly, the primary objective of this study was to assess community knowledge, attitudes, and practices related to rabies disease and dog ownership practices within the Loiyangalani pastoralist communities. Additionally, we aimed to explore the relationship between rabies knowledge and responsible dog ownership practices to identify actionable knowledge gaps to inform tailored, culturally appropriate rabies prevention and dog population management strategies.

## Materials and methods

### Ethical considerations

Ethical approval for this study was obtained from the County Government of Marsabit, Department of Agriculture, Livestock and Fisheries Development, Directorate of Veterinary Services, under reference number MBT/COU/VS/VOL.1/10/2022. Authorisation to engage with the community was also secured from local administrative authorities in Loiyangalani, including the chief, ward administrator, and village elders. Before participation, all respondents received an oral explanation of the study’s purpose, their rights as participants, and assurances regarding the confidentiality of their responses. Only individuals who gave verbal informed consent were interviewed. For participants under 18, verbal consent was obtained from a parent or legal guardian. Written consent was not pursued, as both prior field experience and consultations with local authorities indicated that requesting written signatures often led to mistrust and misinterpretation. Specifically, it creates unrealistic expectations of financial compensation among participants and sparks tension within the broader community, particularly among those who were not selected for the study. To maintain ethical integrity and community cohesion, verbal consent was deemed the most culturally appropriate and respectful approach.

### Study site

The study was conducted in Loiyangalani Town, Marsabit County, a remote and arid region in northern Kenya, located approximately 570 km from Nairobi, Kenya’s capital city. Although agricultural production in Marsabit County includes diverse activities, livestock keeping is the main economic activity in the County with approximately 81% of the population engaged in pastoralism as their main livelihood strategy (12).

### Sampling framework

In May 2023, a comprehensive household census was conducted across the 15 target villages, establishing the most current sampling frame. This census identified 2,030 households within the study area. The minimum required sample size for the study was determined using the formula for estimating a population proportion, assuming a 95% confidence level, a desired precision (or margin of error) of 5%. The calculated sample size was then inflated by 10% to account for potential non-response. Based on this calculation, the target sample size was 411 households. This sample size was deemed sufficient to achieve adequate statistical power for the primary analyses.

A proportionate stratified random sampling approach was used to ensure equitable representation from each of the 15 villages. Within each village, recruitment followed a systematic two-step procedure involving random household selection from village census lists followed by informed verbal consent. Non-participating or unavailable households were systematically replaced by the next household on the randomised list to mitigate non-response bias while maintaining randomisation principles. To maximise data on dog management practices, the study purposively included all consenting dog owners encountered, supplementing the randomly selected sample for this critical population subgroup. Household visits were facilitated by local animal health officers and Community Disease Reporters (CDRs). One adult respondent per household was interviewed, preferably the individual most responsible for dog care in households with dogs. The inclusion criteria required participants to be capable of providing informed verbal consent.

### Data collection

Data was collected using a structured questionnaire, whose development was guided by previously validated KAP survey tools employed in rabies-endemic regions (8, 13, 14). The questionnaire was initially developed in English and translated into Swahili to ensure accessibility and clarity for local enumerators, drawn from the CDR network in Loiyangalani. Prior to deployment, the tool underwent pre-testing to assess clarity, cultural relevance, and contextual appropriateness. Following refinement based on pilot feedback, the final version of the questionnaire was digitised and programmed into the Open Data Kit (ODK) platform for electronic data collection. The CDR enumerators participated in a structured training session covering study questions, ethical procedures, digital data capture, and respondent engagement techniques. The questionnaire encompassed five thematic areas:

- Socio-demographic characteristics.
- Rabies knowledge and awareness, including transmission routes, symptoms in humans and animals, and prevention measures.
- Dog ownership and management practices, such as vaccination, sterilisation, feeding, and roaming behaviour.
- History of animal bites and post-exposure actions.
- Willingness to pay for animal health services, including rabies vaccination and sterilisation.

All responses were digitally recorded using the ODK platform and securely uploaded to a centralised server for data cleaning and analysis, in accordance with established protocols for mobile-based public health surveys (15).

### Data analysis

#### Statistical analysis

Data were analysed using R version 4.2.0 (R Foundation for Statistical Computing, Vienna, Austria). Initially we calculated descriptive statistics, including frequencies, percentages and medians for categorical variables. The distribution of knowledge scores, responsible dog ownership scores, and their demographic association were examined to characterise the overall pattern of knowledge and practices in the study population. However, the geographical variable, village, was excluded from the inferential analysis due to limited variability in individual-level scores and small sample sizes within villages, which introduced statistical instability. Village-level trends were therefore presented descriptively but not included in inferential models.

Bivariate associations between explanatory variables and outcomes (adequate knowledge and responsible dog ownership which are explained in subsequent sections) were assessed using chi-square tests for categorical variables. Simple logistic regression models were then used to calculate unadjusted odds ratios (OR) with 95% confidence intervals for each potential predictor variable. For multivariate analysis, we followed a stepwise approach as recommended for epidemiological studies. Variables with *p* ≤ 0.20 in univariate analysis were considered candidates for inclusion in the multivariate models. We started with the most significant variable and added others sequentially, using likelihood ratio tests (*p* < 0.05 as the threshold) to determine whether each additional variable significantly improved the model. The final models retained only those variables that remained statistically significant after adjustment for other factors. Interaction terms between the final retained predictors were assessed but were not found to be statistically significant and did not improve the models’ fit or discriminatory power. Adjusted odds ratios (aOR) with 95% confidence intervals were calculated from the final multivariate models. Model fit was assessed using the Hosmer–Lemeshow goodness-of-fit test, with *p* > 0.05 indicating adequate fit. The discriminatory power of the models was evaluated using the area under the receiver operating characteristic (ROC) curve (AUC), with values >0.7 considered acceptable. Five-fold cross-validation was performed to assess model stability and generalisability. All statistical tests were two-sided, with *p* < 0.05 considered statistically significant.

In addition, we utilised two standardised composite scoring systems to quantify the relationships between knowledge and practices in rabies prevention, reflecting both theoretical understanding and practical implementation of rabies control measures within pastoralist communities. More specifically, we used the Rabies Knowledge Score (RKS) and the Responsible Dog Ownership Score (RDOS) following methodologies employed in prior studies (14, 16). These composite scores were subsequently analysed to explore patterns and associations in knowledge and behaviour across demographic subgroups.

#### Rabies knowledge score

The Rabies knowledge score, relying on a 5-point scale (0–5) was used to evaluate essential aspects of rabies comprehension vital for disease prevention. Each component was evaluated using a dichotomous scoring system (1 for demonstrated knowledge, 0 for absent knowledge) across five key areas: recognition of rabies as a disease transmitted by animal bites, understanding vaccination as a preventive measure, knowledge of transmission routes, identification of clinical symptoms in humans, and recognition of at least two clinical signs in dogs. A threshold score of ≥3 was used to classify respondents as having “adequate knowledge,” reflecting a foundational level of understanding necessary for effective rabies prevention beyond superficial or incidental awareness.

#### Responsible dog ownership score

The Responsible Dog Ownership Score was used to assess the practical application of evidence-based dog care practices. This five-point scale (0–5) evaluated specific behaviours such as rabies vaccination, regular deworming, appropriate confinement practices, adequate nutrition via food preparation, and reproductive control through castration of males. The threshold for responsible ownership (≥3 criteria) was established to reflect achievable baseline care standards within resource limitations of pastoralist communities where dog management practices vary substantially between social groups based on livelihood utility rather than standardised veterinary models (17, 18). Dog ownership practices in these settings are fundamentally shaped by community-specific requirements, with significant cultural variation in human-dog relationships across different communities (19) and pastoralists typically adapting behaviours observed in peers rather than following formal guidelines (17). This threshold balanced epidemiological necessity with cultural feasibility, ensuring the score could identify sustainable practices transmissible through traditional knowledge networks while representing meaningful progress toward comprehensive dog health management.

## Results

### Respondent demographics

**Figure 1:**
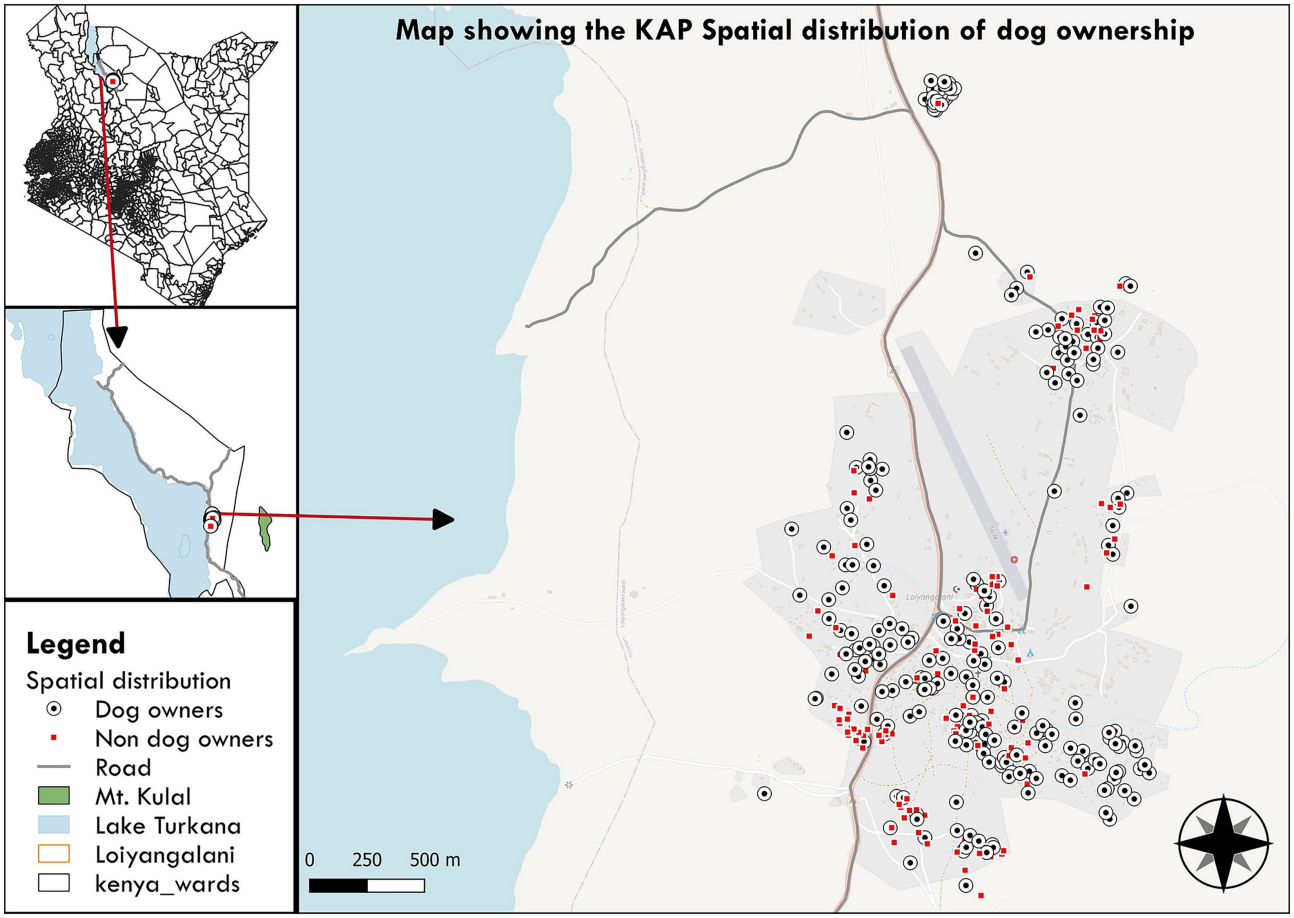
Spatial distribution of dog ownership among study participants in the Loiyangalani area, Marsabit County, Kenya. Black circles with a central dot indicate participants who are dog owners, while red dots represent participants without dogs.

A total of 411 individuals participated in the survey across 15 villages (Figure 1). The majority of respondents were aged 31–50 years (47%) and female (84%). Village participation was determined through proportional sampling based on household numbers (Table 1).

**Table 1:** Socio-demographic characteristics of respondents (*N* = 411).

Characteristic	Category	Frequency (%)
Age Group	Under 18 years	11 (3%)
	18–30 years	154 (38%)
	31–50 years	195 (47%)
	Over 51 years	51 (12%)
Gender	Female	344 (84%)
	Male	67 (16%)
Village	Kiwanja	51 (12%)
	Leatono	41 (10%)
	Kilimambogo	31 (8%)
	Other villages	258 (63%)
	Soweto	15 (4%)
	Nahagan	15 (4%)

### Dog ownership and health management practices

Nearly two-thirds of households (64%) owned dogs, with an average of 1.82 dogs per household. Most dogs were acquired from neighbours (82%) and allowed to roam freely (89%), with minimal restraint practices observed (Table 2).

**Table 2:** Dog ownership practices (*N* = 411).

Characteristic	Category	Frequency (%)
Dog ownership	Own dogs	261(64%)
	Do not own dogs	150 (36%)
Source of dogs (*n* = 261)	From neighbour	213 (82%)
	Other sources	48 (18%)
Dog movement (*n* = 261)	Free roaming	232 (89%)
	Always restrained	20 (8%)
	Other/mixed	9 (3%)

Among dog owners surveyed in this investigation, dog health management was notably poor, with only 22% of dogs vaccinated against rabies and 98% never dewormed. Despite these gaps, only 28% of owners expressed willingness to pay for preventive veterinary services. Most owners (65%) cooked food specifically for their dogs rather than allowing them to scavenge for food (Table 3).

**Table 3:** Dog health and management practices (*N* = 261).

Practice	Category	Frequency (%)
Rabies vaccination	Vaccinated	58 (22%)
	Not vaccinated	203 (78%)
Deworming	Dewormed	5 (2%)
	Not dewormed	256 (98%)
Willingness to pay for preventive services	Willing to pay	74 (28%)
	Not willing to pay	187 (72%)
Dog feeding practices	Cook food specifically	169 (65%)
	Allow free roaming for food	92 (35%)

### Dog reproduction dynamics and dog owners’ fertility regulation practices

High reproductive rates were evident, with 92% of dog-owning households reporting two or more litters annually and 69% reporting litters of six or more puppies. Traditional castration practices – often undertaken without veterinary oversight, anaesthesia, or proper post-operative care – were widespread (83%), with less than half of the respondents who owned dogs (44%) were willing to pay for professional sterilisation services (Table 4).

**Table 4:** Dog reproduction and fertility control practices (*N* = 261).

Characteristic	Category	Frequency (%)
Litters per year	One litter	22 (8%)
	Two litters	136 (52%)
	Three litters	103 (40%)
Average number of puppies per litter	1–2 puppies	30 (12%)
	3–5 puppies	52 (19%)
	6 + puppies	179 (69%)
Traditional castration	Performed	217 (83%)
	Not performed	44 (17%)
Willingness to pay for professional sterilization	Willing to pay	116 (44%)
	Not willing to pay	145 (56%)

### Animal bite incidence and post-bite management

Over one-third of all respondents, (35%) reported experiencing animal bites, with children and adolescents disproportionately affected (47% of bite victims were under 18 years). Wildlife accounted for the majority of bites (73%) followed by dogs (25%) (Table 5).

**Table 5:** Animal bite incidence and characteristics (*N* = 411).

Characteristic	Category	Frequency (%)
Animal bite experience	Bitten	142 (35%)
	Not bitten	269 (65%)

Among those bitten (*N* = 142)		
Gender of bite victims	Female	74 (52%)
	Male	68 (48%)
Age of bite victims	Under 18 years	67 (47%)
	18–30 years	37 (26%)
	31–50 years	28 (20%)
	Over 51 years	10 (7%)
Biting species	Wildlife	103 (73%)
	Dogs	35 (25%)
	Cats	4 (3%)

Health-seeking behaviour following bites showed a mixed pattern among the respondents included in the study. While 66% sought professional medical care, with 82% of these receiving post-exposure prophylaxis, many also utilised traditional treatments including herbal remedies (30%). Notably, only 23% of those seeking formal care reported received adequate wound management (Table 6).

**Table 6:** Health-seeking behaviour following animal bites (*N* = 142).

Treatment type	Frequency (%)
Professional medical care*	
Received PEP	76 (82%)
Received wound management	21 (23%)
Traditional/alternative methods*	
Traditional herbs	42 (30%)
Soap and water washing	9 (6%)
Self-medication	8 (6%)
Traditional healer consultation	6 (4%)

*Percentages calculated from those who sought professional medical care (*n* = 93).

### Demographic patterns in rabies knowledge and responsible dog ownership

The overall Rabies Knowledge Score among participants was notably high (354/411, 86.1%). The demographic analysis revealed the patterns of rabies knowledge among participants (Table 7). Gender differences were modest for adequate rabies knowledge, with males achieving 91.0% adequate knowledge compared to 85.2% for females (*p* = 0.2807), while both groups maintained similar median knowledge scores of 4 out of 5. However, gender disparities were more pronounced for responsible dog ownership practices, where males demonstrated higher compliance rates (28.3% vs. 16.3%, *p* = 0.073) despite equivalent median ownership scores of 2.0.

**Table 7:** Demographic patterns of rabies knowledge and responsible dog ownership practices.

Variable	Categories	Rabies knowledge (all participants, *n* = 411)						Responsible dog ownership (dog owners, *n* = 261)					
		Median	Yes	No	Total	% Adequate	*p*-value	Median	Yes	No	Total	% Responsible	*p*-value
Gender	Male	4	61	6	67	91.0%	0.2807	2.0	15	38	53	28.3%	0.0730
	Female	4	293	51	344	85.2%		2.0	34	174	208	16.3%	
Age Group	<18	4	10	1	11	90.9%	0.5931	2.0	3	7	10	30.0%	0.5105
	18–30	4	130	24	154	84.4%		2.0	12	71	83	14.5%	
	31–50	4	172	23	195	88.2%		2.0	30	114	144	20.8%	
	>50	4	42	9	51	82.4%		1.5	4	20	24	16.7%	
Village	Kiwanja	5	49	2	51	96.1%	0.5397	2.0	11	23	34	32.4%	0.0089
	Nawoitorong	5	19	1	20	95.0%		2.0	3	4	7	42.9%	
	Kilimambogo	4	28	3	31	90.3%		2.0	7	13	20	35.0%	
	Kulapesa	4	25	3	28	89.3%		2.0	2	19	21	9.5%	
	Kulasamaki	4	25	3	28	89.3%		1.0	2	21	23	8.7%	
	Kulamawe	4	26	4	30	86.7%		2.0	6	15	21	28.6%	
	Nahagan	5	13	2	15	86.7%		2.0	3	4	7	42.9%	
	Soweto	5	13	2	15	86.7%		2.0	1	5	6	16.7%	
	Achukulee	4	24	4	28	85.7%		1.0	1	6	7	14.3%	
	St. Martin	4	20	4	24	83.3%		2.0	3	16	19	15.8%	
	Nakwamekui	4	19	4	23	82.6%		1.0	0	9	9	0.0%	
	Town	5	19	4	23	82.6%		2.0	4	6	10	40.0%	
	Dikilkimat	4	20	5	25	80.0%		2.0	0	11	11	0.0%	
	Nawapa	4	23	6	29	79.3%		2.0	4	25	29	13.8%	
	Leatono	4	31	10	41	75.6%		1.0	2	35	37	5.4%	

N/B: Median: Median score on 0–5 scale for each outcome; Adequate Knowledge: Score ≥3 out of 5 on rabies knowledge assessment; Responsible Ownership: Score ≥3 out of 5 on ownership practices; *p*-values from chi-square tests comparing demographic groups; Villages ranked by % adequate knowledge (highest to lowest).

Age-related patterns showed relatively stable knowledge levels across groups, ranging from 82.4% adequate knowledge in participants >50 years to 90.9% in those <18 years (*p* = 0.5931). Median knowledge scores remained consistently at 4 across all age categories. For responsible ownership, younger participants (<18 years) showed the highest compliance at 30.0%, while the 18–30 age group had the lowest at 14.5% (*p* = 0.5105). Notably, participants >50 years demonstrated the lowest median ownership score of 1.5, compared to 2.0 across other age groups.

Village-level variations revealed substantial heterogeneity in both outcomes. Knowledge adequacy ranged from 75.6% in Leatono to 96.1% in Kiwanja (*p* = 0.5397), with median scores varying from 4 to 5 across villages. More striking disparities emerged for responsible ownership, where village-level compliance varied dramatically from 0% in Dikilkimat and Nakwamekui to 42.9% in both Nahagan and Nawoitorong (*p* = 0.0089). Despite this wide range in compliance rates, median ownership scores showed limited variation (1.0–2.0 across villages). Village analysis revealed a geographic clustering pattern, with several villages (Kiwanja, Nawoitorong, Kilimambogo) exhibiting both high knowledge levels (>90% adequate) and relatively high responsible dog ownership practices (>30% responsible). Conversely, villages like Leatono, Kulasamaki, and Nakwamekui demonstrated a combination of lower knowledge adequacy and low responsible dog ownership practices.

### Predictors of adequate rabies knowledge

In the univariate analysis, willingness to pay for dog sterilisation surgery was significantly associated with adequate rabies knowledge (OR 2.95, 95% CI: 1.33–7.22, *p* = 0.0110). A similar trend was observed for willingness to pay for vaccination or deworming services (OR 1.96, 95% CI: 0.92–4.47, *p* = 0.0924), although this did not reach statistical significance. Other variables, including gender, age group, dog ownership status, and history of being bitten by an animal, showed no significant associations with knowledge adequacy (Table 8). In the final multivariate logistic regression model, only willingness to pay for surgical sterilisation remained an important predictor of adequate knowledge (aOR 2.95, 95% CI: 1.33–7.22, *p* = 0.0110). The model demonstrated fair discriminatory performance, with an area under the curve (AUC) of 0.632 under five-fold cross-validation and a mean classification accuracy of 86.8% [± 4.4% Standard Deviation (SD)].

**Table 8:** Analysis of factors associated with adequate rabies knowledge.

Variable	Categories	Yes	No	Total	OR (95% CI)	*p* value	aOR (95% CI)
Gender	Male	61	6	67	Reference		
	Female	293	51	344	0.57 (0.21–1.28)	0.2085	
Age group	<18	10	1	11	Reference		
	18–30	130	24	154	0.54 (0.03–3.03)	0.5673	
	31–50	172	23	195	0.75 (0.04–4.18)	0.7863	
	>50	42	9	51	0.47 (0.02–2.94)	0.4927	
Owns a dog	No	127	23	150	Reference		
	Yes	227	34	261	1.21 (0.68–2.13)	0.5152	
Previously bitten	No	235	34	269	Reference		
	Yes	119	23	142	0.75 (0.42–1.34)	0.3221	
Willing to pay for vaccination/deworming	No	125	24	149	Reference		
	Yes	102	10	112	1.96 (0.92–4.47)	0.0924	
Pay surgery	No	119	26	145	Reference		Reference
	Yes	108	8	116	2.95 (1.33–7.22)	**0.0110**	2.95 (1.33–7.22)

OR, odds ratio; aOR, adjusted odds ratio (from the multivariate model); CI, confidence interval. Adequate knowledge: knowledge score ≥3 out of 5 domains. The bold values indicate significance (*p* < 0.05).

### Predictors of responsible dog ownership practices

In the univariate analysis, gender demonstrated the strongest association with responsible dog ownership. Female respondents had significantly lower odds of meeting responsible dog ownership criteria compared to males (OR 0.50, 95% CI: 0.25–1.02, *p* = 0.0495). Other variables, including previous bite experience (OR 1.84, *p* = 0.0589), willingness to pay for dog sterilisation (OR 1.70, *p* = 0.0978), and willingness to pay for vaccination/deworming (OR 1.66, *p* = 0.1133), showed trends toward significance but did not reach the conventional threshold. Adequate rabies knowledge was not associated with responsible dog ownership (OR 1.09, 95% CI: 0.45–3.06, *p* = 0.8569), suggesting a possible disconnect between knowledge and practice.

In the final multivariate logistic regression model (Table 9), only gender remained a statistically significant predictor. Female respondents had lower adjusted odds of meeting responsible dog ownership criteria than their male counterparts (aOR 0.50, 95% CI: 0.25–1.02, *p* = 0.0495). The model demonstrated moderate discriminatory capacity, with an area under the curve (AUC) of 0.56 under cross-validation and a mean classification accuracy of 81.2% (± 4.5% SD).

**Table 9:** Analysis of factors associated with responsible dog ownership.

Variable	Categories	Yes	No	Total	OR (95% CI)	*p* value	aOR (95% CI)
Gender	Male	15	38	53	Reference		Reference
	Female	34	174	208	0.50 (0.25–1.02)	**0.0495**	0.50 (0.25–1.02)
Age group	<18	3	7	10	Reference		
	18–30	12	71	83	0.39 (0.09–2.02)	0.2192	
	31–50	30	114	144	0.61 (0.16–2.98)	0.4981	
	>50	4	20	24	0.47 (0.08–2.85)	0.3870	
Previously bitten	No	27	147	174	Reference		
	Yes	22	65	87	1.84 (0.97–3.47)	**0.0589**	
Willing to pay for vaccination/deworming	No	23	126	149	Reference		
	Yes	26	86	112	1.66 (0.89–3.11)	0.1133	
Pay dog surgery	No	22	123	145	Reference		
	Yes	27	89	116	1.70 (0.91–3.19)	0.0978	
Adequate-rabies knowledge	No	6	28	34	Reference		
	Yes	43	184	227	1.09 (0.45–3.06)	0.8569	

OR, odds ratio; aOR, adjusted odds ratio (from the multivariate model); CI, confidence interval. Responsible Dog Ownership, Meeting at least 3 of 5 criteria (vaccination, deworming, confinement, feeding, castration). The bold values indicate significance (*p* < 0.05).

### Knowledge-practice gaps

Among the 227 of the 261 dog owners with adequate rabies knowledge, only 50 (22%) had vaccinated their dogs against rabies, revealing a 78 percentage point gap between knowledge and practice. Other key practices were similarly limited: only four owners (1.8%) reported regular deworming, and 17 (7.5%) always confined their dogs. Despite their adequate knowledge, less than half of these owners expressed a willingness to pay for preventive services. Specifically, only 102 (45%) of dog owners surveyed in this study (*n = 261*) were willing to pay for vaccination and deworming services; similarly, only 108 respondents who owned dogs (48%) were willing to pay for surgical sterilisation.

## Discussion

This study offers a community-level evaluation of rabies-related knowledge, attitudes, and dog ownership practices in a remote pastoralist region of northern Kenya. A strong baseline awareness of rabies was observed, with 86.1% of all 411 respondents and 87.0% of the 261 dog owners demonstrating adequate knowledge across essential components, including transmission routes, clinical signs in animals and humans, and preventive measures such as vaccination.

However, this high level of awareness was not matched by corresponding behavioural practices. In evidence of this fact, only 18.8% of dog owners fulfilled the criteria for responsible ownership. Among those with adequate knowledge, rabies vaccination coverage remained low at just 22%, which is below the WHO’s recommended 70% threshold necessary to interrupt disease transmission and achieve effective control (1).

Similar knowledge-practice gaps have been documented in other endemic settings. For example, in a Kenyan context, Obara et al. (20) in 2025 observed that despite high rabies awareness, practical vaccination adherence was hindered by limited public awareness, insufficient funding, and infrastructural constraints. This mirrors findings from Turkana, Kenya, where veterinary professionals perceived insufficient awareness, lack of information regarding immunisation campaigns, and vaccination costs as primary obstacles to dog vaccination (21). Further emphasising these barriers, another recent study on rabies elimination challenges in Lamu County, Kenya, highlighted persistent stockouts of human rabies vaccines and a lack of awareness of bite wound management at health facilities, indicating issues with access to vital rabies prevention services (22). Beyond Kenya, a recent study in Zambia explicitly discussed the challenges of achieving herd immunity in dogs, including difficulties in pre-campaign community sensitisation (23). Similarly, although not in Africa, a recent study in Kazakhstan also noted that high rabies awareness did not always translate into appropriate practices, identifying factors such as free grazing and improper carcass disposal as risk factors for rabies (15). These consistent findings across various settings underscore the need for interventions that improve awareness and address barriers to implementation, including social, economic, and geographical constraints that hinder access to veterinary services. Gender was the only significant predictor of responsible dog ownership, with female respondents having significantly lower adjusted odds compared to males. This finding must be interpreted within the sociocultural and economic structure of the pastoralist household. While women were the primary respondents (84%) and are often daily caregivers, the specific tasks that constitute “responsible dog ownership” in this score such as paying for veterinary services, procuring vaccines (22% coverage), or arranging professional sterilization (44% willing to pay) often fall under the purview of male decision-makers responsible for household finances and external services. The lower odds for females may therefore reflect a constraint in decision-making power and financial access rather than a lower commitment to dog welfare. The results reported here and those from other studies reinforce the critical need for comprehensive interventions that address not only knowledge deficits but also the socio-economic and logistical hurdles to effective rabies control (24, 25).

In addition to the findings associated with the level of rabies knowledge, this study highlights the widespread reliance on traditional methods for managing dog reproduction and health, reflecting both cultural norms and structural barriers to formal veterinary care. Most dog owners in the study reported using traditional castration techniques, which are often performed by a community member. There is limited publication of this practice, however, broader context of traditional animal management, where formal animal health services are scarce, suggests communities often resort to informal methods that raise significant animal welfare concerns regarding infection risk and unintended behavioural consequences (26). In our investigation, only 44% of dog owners expressed willingness to pay for surgical sterilisation, with the willingness to pay being higher among those with adequate rabies knowledge. These findings suggest that knowledge may shape intent, but socioeconomic factors ultimately determine action.

For example, a study in North Western Ethiopia found that while a large majority of dog owners had a positive intention to control rabies through vaccination, their willingness to pay for the vaccine decreased as the price increased, and income was a significant factor in actual willingness to pay (27). Bridging this gap will require more than clinical service availability. It will necessitate mobile, community-based delivery models; culturally sensitive messaging; and potentially subsidised or incentivised service options. Strengthening the role of local animal health officers and Community Disease Reporters (CDRs) in providing frontline education and linking households to veterinary services could be a key strategy for improving dog welfare and rabies prevention in these settings, a strategy supported by organisations like Vétérinaires Sans Frontières (VSF) through their work with Community Animal Health Workers and demonstrated by successful community-based surveillance systems in other pastoral communities in Sub-Saharan Africa (28).

The study also revealed a mixed and often inadequate pattern of health-seeking behaviour among respondents who experienced animal bites. While most respondents (66%) sought professional medical care, a substantial proportion either failed to access formal care or relied exclusively on alternative treatments. This finding aligns with other studies, such as those in Nigeria, where a significant percentage of dog bite victims preferred or resorted to traditional remedies. Even among those who reached health facilities, only 82% received PEP, and just 23% reported receiving basic wound management (29). This low uptake of critical care is reported in studies from South Africa, which found that only 27% of rabies cases received PEP (30), and in an Indian rural community, where only 26% knew the correct first aid procedures (31). These findings are concerning given the urgency of timely and appropriate post-bite response in rabies prevention. The underutilisation of PEP and low uptake of wound care may reflect a lack of awareness, limited access to PEP vaccines, or reliance on cultural beliefs about treatment (32). This highlights a need for public health messaging that not only educates communities about rabies risk but also clearly communicates the steps to take following an animal bite, particularly the importance of immediate wound cleaning and prompt medical attention. Research consistently supports that improving public education, enhancing access to medical care, and even involving and incentivising traditional healers to refer dog bite cases to health centres can strengthen rabies prevention and control programs (4). In pastoralist settings where access to health services may be constrained, expanding the role of community health workers and training local providers to recognise and respond effectively to bite incidents could improve treatment outcomes and reduce the likelihood of rabies-related mortality, a strategy endorsed by the WHO’s updated guidelines advocating for PEP delivery and monitoring via task sharing involving non-specialist health workers (33).

### Conclusions and recommendations

This study highlights the complex interplay between rabies knowledge, dog ownership practices, and health-seeking behaviour in a remote, pastoralist community in northern Kenya. While rabies knowledge levels were high, corresponding preventive practices such as dog vaccination, confinement, deworming, and appropriate post-bite response were substantially lacking. The observed knowledge–practice gap highlights the limitations of awareness-raising alone. It underscores the need for interventions that address economic, cultural, and systemic barriers to responsible dog ownership and rabies prevention. The documented knowledge-practice gap (86.1% knowledge vs. 22% vaccination coverage) in this high-risk pastoralist region provides critical baseline data essential for Kenya’s commitment to the “Zero by 30” global rabies elimination goal. Specifically, these findings quantify the priority barriers economic constraint, access, and poor post-bite management that must be overcome with tailored, subsidised, and community-based strategies to achieve the 70% vaccination coverage required to eliminate human rabies deaths in this region by 2030.

Key recommendations emerging from this study include the development of integrated, community-based rabies control strategies that are tailored to the unique sociocultural and ecological realities of pastoralist populations. These should consist of (i) strengthening access to veterinary services through mobile clinics, (ii) subsidised sterilisation and (iii) subsidised vaccination campaigns. Furthermore, efforts need to be made to enhance community engagement by leveraging trusted local actors and tailoring education campaigns to address not just knowledge, but beliefs, motivations, and resource constraints. For example, future campaigns could endeavour to address gender-based disparities by ensuring that both men and women are actively involved in decision-making around dog care and have equal access to information and services. In addition, improving post-bite care awareness could be facilitated through coordinated health communication that emphasises immediate wound cleaning and prompt presentation at health facilities.

The KAP study findings presented here will guide the development of tailored risk communication strategies, support community engagement, and inform the design of integrated rabies prevention and dog population management programs that are responsive to the sociocultural and ecological context of this pastoralist setting.

### Limitations of the study

This study has several limitations that should be considered when interpreting the findings. First, the cross-sectional design limits the ability to infer causality between knowledge, attitudes, and practices. While associations were identified, temporal relationships cannot be established. Second, the data relied on self-reported responses, which may be subject to social desirability bias or recall inaccuracies, particularly regarding topics such as animal bite history, vaccination status, and traditional treatment practices. Third, although the study employed a proportionate stratified random sampling strategy, some villages contributed relatively small numbers of respondents. This limited the statistical power to include the village-level variable in inferential analyses, despite observed descriptive differences. Consequently, potentially important geographic patterns could not be examined using multivariate models. Fourth, the sampling strategy resulted in a high proportion of female respondents (84%). This is often observed in household surveys in pastoralist settings where male heads are mobile with livestock. While females were the primary respondents and provided rich data on dog care, this imbalance may introduce selection bias and affect the interpretation of gender-specific practices. Specifically, in this cultural context, roles such as securing vaccinations or performing castration (elements of the Responsible Dog Ownership Score) may be traditionally designated to men, which could artificially lower the compliance rates reported by female respondents. Despite these limitations, the study offers valuable insights into community-level dynamics of rabies knowledge and dog management practices in a highly underserved pastoralist region, and it provides a strong foundation for designing tailored, community-based interventions.

## Data Availability

The raw data supporting the conclusions of this article will be made available by the authors, without undue reservation.
